# Two Cases of Bronchocele

**Published:** 1835-04-01

**Authors:** James Dameram


### *Two Cases of Bronchocele, by* James Dameram, Esq.

We have been favoured, by Dr. Henry Davies, with the papers of a deceased medical officer, Staff assistant-Surgeon Dameram, together with permission to extract what we pleased. We shall avail ourselves of our privilege, by selecting, from the records of this diligent and meritorious officer, two cases of bronchocele, the interest of which depends on the success of a very bold method of treatment. They are to be found in the author's report of cases occurring in the detachment hospital at Penetanguishene, Upper Canada, in the quarter ending 31st March, 1833. Mr. Dameram observes, that eighteen cases of bronchocele had occurred since the 1st of January; a number so great as to make the disease appear epidemic, for it was in the proportion of two cases to five men. The disease was not endemic, and the detachment had been at this post eighteen months, without being attacked by it.

The following are the cases just referred to: for the method of treating them our author confesses himself indebted to Dr. Shortt, of the 79th Regiment. [Edit. *Med. Q. R.*]

Robert Morton, admitted 8th February.—℞. Iodin. ʒss.; Conf. Rosæ, q. s. ut fiant pil. lx. quarum capiat j. nocte maneque, ante jentaculum, et post cænam, unâ horâ interpositâ. —℞. Iodin. ʒj.; Adipis suillæ, ℥j. M. fiat unguent. nocte maneque applicandum.

12th. A good deal of redness and irritation is produced by the ointment.—Pt. in usu unguenti.

13th, vespere. Empl. Lyttæ tumori.

14th. The blistered part has risen well, and the discharge of serum is copious.—Ung. Antim. Tart. applicetur parti vesicatæ.

15th. The application of the tartar-emetic ointment was attended with considerable pain and irritation. The cutis has partially sloughed, the remaining part being covered with pustules.—Applicetur Ung. Cetacei parti ulceratæ. Pt. in usu pilularum.

17th to 21st. The sloughed parts are healing kindly, and there is certainly a diminution in the size of the tumour.

From this time to the middle of the next month, the man took 1½ grains of iodine a day, with occasionally a purge, without the slightest change in the appearance of the neck. I then determined on a second trial of the counter-irritation, which was performed on the 19th of March, in the same way as before, having previously, for four days, rubbed in a scruple of the iodine ointment night and morning. Precisely the same results followed; the same irritation, same sloughing. He still continued his pills.

24th. The healing of the parts now allows me to see that again a very sensible diminution of the tumour has taken place.

27th. The tumour is still decreasing; and so it went on, till there appeared to be nothing left but a little fullness in the parts. The sterno-cleido-mastoidei are fully developed, as are also the rings of the trachea and thyroid cartilage.

On the 6th of April, I discharged this man.

Rod. M'Leod, the other man, was admitted at the same time with Morton, and treated like him in every respect, and on the same days; with this exception, that for three weeks and a half he took but one grain of iodine a day, because I suspected an organic disease of the heart, or commencement of the aorta; and I feared the iodine at first, especially as he has had, since I have been here, during a warm day, a momentary attack of angina pectoris and syncope: however, the iodine, guarded by purgatives occasionally, afterwards agreed very well with him. The results with him were precisely the same as in Morton's case, during treatment; and now his tumour is, I think, even more completely gone, in the middle and left side of the throat; but, on the right side, there is rather more fullness than in the other case. He was discharged on the same day with Morton, and I shall carefully watch them.

